# Survey of Professionals of the European Public Health Association (EUPHA) towards Direct-to-Consumer Genetic Testing

**DOI:** 10.1093/eurpub/ckac139

**Published:** 2022-09-30

**Authors:** Flavia Beccia, Ilda Hoxhaj, Michele Sassano, Jovana Stojanovic, Anna Acampora, Roberta Pastorino, Stefania Boccia

**Affiliations:** Department of Health Science and Public Health, Università Cattolica del Sacro Cuore, Rome, Italy; Department of Health Science and Public Health, Università Cattolica del Sacro Cuore, Rome, Italy; Department of Surgery Oncology and Gastroenterology, University of Padova, Padova, Italy; Department of Health Science and Public Health, Università Cattolica del Sacro Cuore, Rome, Italy; Department of Medical and Surgical Sciences, University of Bologna, Bologna, Italy; Department of Health Science and Public Health, Università Cattolica del Sacro Cuore, Rome, Italy; Department of Health, Kinesiology, and Applied Physiology (HKAP), Concordia University, Montreal, Quebec, Canada; Montreal Behavioural Medicine Centre, CIUSSS du Nord-de-l'Île-de-Montréal, Montréal, Québec, Canada; Department of Health Science and Public Health, Università Cattolica del Sacro Cuore, Rome, Italy; Department of epidemiology, Regional Health System, Rome, Italy; Department of Health Science and Public Health, Università Cattolica del Sacro Cuore, Rome, Italy; Department of Woman and Child Health and Public Health, Fondazione Policlinico Universitario A. Gemelli IRCCS, Rome, Italy; Department of Health Science and Public Health, Università Cattolica del Sacro Cuore, Rome, Italy; Department of Woman and Child Health and Public Health, Fondazione Policlinico Universitario A. Gemelli IRCCS, Rome, Italy

## Abstract

**Background:**

The increasing availability of Direct-to-Consumer Genetic Tests (DTC-GTs) has great implications for public health (PH) and requires literate healthcare professionals to address the challenges they pose. We designed and conducted a survey to assess the state of knowledge, attitudes and behaviours of PH professionals members of the European Public Health Association (EUPHA) towards DTC-GTs.

**Methods:**

EUPHA members were invited to participate and fill in the survey. We performed multivariable logistic regression to evaluate associations between selected covariates and knowledge, attitudes and behaviours of healthcare professionals towards DTC-GT.

**Results:**

Three hundred and two professionals completed the survey, 66.9% of whom were not involved in genetics or genomics within their professional activities. Although 74.5% of respondents were aware that DTC-GTs could be purchased on the web, most of them reported a low level of awareness towards DTC-GTs applications and regulatory aspects. The majority did not approve the provision of DTC-GTs without consultation of a healthcare professional (91.4%), were doubtful about the test utility and validity (61%) and did not feel prepared to address citizens’ questions (65.6%). Predictors of knowledge on DTC-GT were the involvement in genetics/genomics and receiving training during the studies (*P* < 0.0001 and *P* = 0.043). Predictors of attitudes were medical degree and knowledge about DTC-GTs (*P* = 0.006 and *P* = 0.027).

**Conclusions:**

Our results revealed a high level of awareness of DTC-GT web purchasing and a moderate to low level of awareness towards their applications. Despite the overall positive attitudes, PH professionals reported a high need for strengthening regulatory aspects of DTC-GTs provision process.

## Introduction

Following the completion of the Human Genome Project, the past years have consequently witnessed enormous discoveries in the field of genomics, with fundamental implications for public health (PH).^1^ The Public Health Genomics European Network identified, in 2006 as a key issue the education in Public Health Genomics (PHG) for professionals in PH.^2^ Fifteen years later, however, a low level of knowledge, alongside with the need to increase awareness on genomics, has been reported in a recent survey of PH professionals at the European level.^3^ With the increased availability of genetic testing, as well as of Direct-to-Consumer Genetic Tests (DTC-GTs), it is of utmost relevance for healthcare to have literate PH professionals on the role of genomics in improving population health.^4^ The provision of DTC-GTs outside an established healthcare setting, without a physician’s prescription, has raised several concerns among policymakers and PH experts, considering the low literacy of genetic and non-genetic healthcare professionals and the increased interest among the general public.^5^ Despite the strong recommendation by the European Society of Human Genetics (ESHG) for the involvement of a health professional in the order, process and interpretation of DTC-GT results,^6^ the level of awareness remains poor, especially among non-genetics health professionals. A systematic review published in 2015, reported an overall low level of awareness and poor confidence among healthcare professionals.^7^ However, to date, no studies specifically addressing the perspectives of PH professionals on DTC-GTs have been published, nor in Europe nor elsewhere. Considering that DTC-GTs are becoming a PH issue, it is crucial to assess and understand PH professionals’ literacy. DTC-GTs policy issues, in particular, PH professionals’ perspectives have been the focus of a European project, the Innovative Partnership for Action Against Cancer (iPAAC) Joint Action.^8^ Within this project, we conducted a survey aiming to evaluate the knowledge, behaviour and attitudes of PH professionals belonging to European Public Health Association (EUPHA) towards DTC-GTs.

## Methods

This research was carried out in two stages. The first one involved a literature review to identify different items to be included in the questionnaire. In a second phase, based on the results of our review, a questionnaire was developed and then, submitted to a panel of experts for validation and final approval before the distribution.

### Phase 1: literature review

The survey was structured on the basis of a literature review. We included surveys designed properly to explore the health professionals’ knowledge, attitudes and behaviours towards DTC-GT, which reported at least one of the items of our interest. The literature search was conducted in PubMed, including articles published until 1 March 2019, without any language restriction, using the search terms: ‘knowledge’, ‘attitudes’, ‘behaviors’, ‘health personnel’, ‘health professional’, ‘direct-to-consumer’, ‘genetic’ and ‘genomic’. Three researchers independently (A.A., M.S. and J.S.) screened the retrieved articles by titles and abstracts, and in a second step evaluated full-text articles for inclusion. Data extracted from the included articles were the following: year of publication, country, type of healthcare professionals involved and the assessed items concerning knowledge, attitudes and/or behaviours about DTC-GTs. Moreover, the references of the included articles were hand searched, in order to retrieve any other additional eligible article.

### Phase 2: Survey development

All the extracted items were further evaluated for inclusion in the questionnaire by three researchers (A.A., M.S. and J.S.) and were adapted to PH professionals. A first initial draft of the questionnaire was developed, and afterwards was validated by means of focus group methodology.^9^ The focus group involved seven Italian experts in the field: three geneticists and four PH professionals, already involved in the iPAAC project activities and affiliated to the Italian National Institute of Health and Università Cattolica del Sacro Cuore. In three online meetings until consensus, the expert panel analysed the structure of the questionnaire, focusing on the number of questions, language, type of questions and answers, ensuring that the survey did not contain confusing or double-barrelled questions. According to their comments, additional changes were implemented, and the final version of the questionnaire was created and distributed.

### Questionnaire description

A specific web-based questionnaire was developed containing 44 questions in five sections (Supplementary file), aiming to explore:

Section 1: demographic characteristics and personal details (five questions);

Section 2: professional activity (seven questions);

Section 3: awareness and knowledge of DTC-GT (ten questions);

Section 4: attitudes and personal opinions about DTC-GT (fourteen questions); and

Section 5: experience and personal behaviour regarding DTC-GT (eight questions).

In detail, awareness and knowledge on DTC-GT were assessed by two yes/no questions asking about: (i) awareness on companies advertising and selling genetic tests directly to consumers, and (ii) awareness on the possibility to purchase DTC-GTs over the web. Furthermore, we assessed the awareness on DTC-GTs applications; professional organizations which issued statement/opinion/recommendation towards DTC-GTs, legal national framework related to DTC-GTs.

Attitudes towards DTC-GTs were assessed asking five-point Likert scale questions about: the personal opinion towards the potential benefits and risks, the provision outside the traditional healthcare setting, the introduction after proven clinical validity and utility, and cost-effectiveness. These attitudes were considered positive when the respondents agreed or strongly agreed with the statements, and negative when the respondents disagreed or strongly disagreed. Other attitudes were assessed using yes/no questions asking about the: accessibility of DTC-GTs information to physicians, involvement of a health professional, regulation on a national level, results’ helpfulness or harmfulness and preparedness to answer citizen’s questions.

Behaviours towards DTC-GTs were assessed asking about the willingness to personally undergo a DTC-GT in the future, the previous personal experience being tested with DTC-GTs, and the experience with citizens that underwent a DTC-GT.

### Data collection and analysis

The web-based questionnaire was distributed through the EUPHA Newsletter on May 2019^10^ and February 2020,^11^ and also through EUPHAnxt Newsletter on December 2019.^12^ Additionally, EUPHA office sent an email to EUPHA Section Presidents, in order to distribute the survey to EUPHA Section Members. The data were collected anonymously between 31 May 2019 and 22 November 2021.

Survey responses were collected in an electronic data sheet. Descriptive analyses were performed using frequencies and percentages for categorical data and mean and standard deviation (SD) for continuous data.

An analysis of determinants of knowledge, attitudes and behaviour towards DTC-GTs was conducted through the development of multivariable logistic regression models. The variables ‘knowledge on DTC-GT’, ‘attitudes on DTC-GT’ and ‘behaviours toward DTC-GT’ originally consisting of multiple categories, were collapsed into two levels, adapting the methodology previously used in other surveys on similar topics^3^^,^^13^^,^^14^: knowledge, attitudes or behaviour were attributed to respondents providing correct responses to the 75% of the questions included. Covariates included in the models were: age, sex, personal or family history of genetic disorder or hereditary syndrome, personal or family history of cancer, involvement in genetic/genomics within professional activities, exposure to information on genetic testing during undergraduate or post-graduate education (with receiving none of them as reference category), area of degree (with non-medicine as reference category) and sector of work (with the non-academic sector as reference category). Multivariable logistic regression models were built using the strategy suggested by Hosmer and Lemeshow. Each variable was examined by univariable analysis and was included in the multivariable logistic model when the *P* values was <0.15. The influence of the independent variables on each binary outcome investigated was expressed as odds ratios (ORs) and 95% confidence interval (CI).

Statistical significance was set at a *P* values <0.05. The statistical analysis was performed using STATA 16.0 software (Stata Corporation, College Station, TX, USA).

Ethical approval for this study was obtained from the *Ethics Committee* of the Policlinico Universitario ‘Agostino *Gemelli*’, Rome.

## Results

### Results of the literature review

We retrieved 56 articles through the initial search and, after screening of titles and abstracts, 22 full-texts were further evaluated (Supplementary figure S1). Of these, 10 articles were eligible and additionally, one study was identified through snowball search, leading to a final number of 11 articles included in our review.^15–25^ Main characteristics and findings of included articles are summarized in Supplementary table S1. The articles were published from 2007 to 2015, most of them were conducted in the USA (46%, *n* = 5)^16^^,^^17^^,^^19^^,^^24^^,^^25^ and Europe (27%).^18^^,^^21^^,^^22^ Six studies (64%) targeted general practitioners (GPs) and/or physicians,^16–21^ four studies (36%) genetic specialists and/or genetic counselors^15^^,^^22^^,^^24^^,^^25^ and one study focused on both.^23^ These articles evaluated (91%) the level of knowledge,^15–24^ the sources of information (40%),^17^^,^^19^^,^^21^^,^^23^ their attitudes (91%)^15–18^^,^^20–25^ and behaviours (82%)^15–19^^,^^21^^,^^22^^,^^24^^,^^25^ as reported in Supplementary material S1.

Based on the information retrieved from the articles included in the review, we extracted a total of 147 questions, 45 regarding knowledge, 67 attitudes and 35 on behaviours. We then checked for duplicates and suitability for inclusion, leading to the removal of 14 questions in the knowledge section, 46 for attitudes and 26 in the behaviour section. These questions were further adapted, and the questionnaire, containing 44 questions, was validated and approved by the focus group of experts (Supplementary material S2).

### Results of the survey

#### Main characteristics of the respondents

Three hundred and two PH professionals completed all sections of the survey, of whom socio-demographic characteristics are reported in table 1.

**Table 1:** Socio-demographic characteristics of respondents

Characteristics	*N*	%
Sex		
Female	167	55.3
Male	135	44.7
Age		
23–38	199	65.89
39–54	70	23.18
55–70	30	9.93
>70	3	0.99
Personal or family history of a genetic disorder or hereditary syndrome	35	11.6
Personal or family history of cancer	141	46.7
Involvement in genetics/genomics within the professional activities	100	33.1
Highest educational degree obtained		
Master	150	49.67
Doctorate	79	26.16
Bachelor	73	24.17
Area of degree		
Medicine	174	57.62
Health professions (nursing, etc.)	27	8.94
Public Health	22	7.28
Biology	21	6.95
Economics	19	6.29
Other (pharmacy, management, computer sciences, statistics, social sciences, etc.)	39	12.92
Information on genetic testing during undergraduate training	173	57.3
Information on genetic testing during postgraduate training	145	48
Sector of work		
Academic/research	149	49.34
Hospital	62	20.53
Public health service (i.e. vaccination service/screening program/maternal-child health service. etc.)	35	11.59
Technical agency	20	6.62
Other (local or national government, primary care, pharmaceutical)	36	11.92
Main professional area		
Statistics and epidemiology	109	16.17
Public health policy	101	14.94
Non-communicable diseases control	71	10.50
Health services research	49	7.25
Health services management	46	6.80
Cancer prevention	44	6.51
Communicable diseases control	41	6.07
Health technology assessment	40	5.92
Other (e.g. health economics, health impact assessment, child and adolescent health, public mental health, etc.)	175	25.9

The mean age was 37.5 years old (SD = 10.98), and 55.3% of the participants were female. The majority of respondents were from European countries, such as Italy (43.71%; *n* = 132), Belgium (7.95%, *n* = 24), UK (4,3%, *n* = 13), Spain (3.64%, *n* = 11), Germany (3.31%, *n* = 10), Switzerland (3.31%, *n* = 10) and the Netherlands (2.98%, *n* = 9). The majority did not have a personal or family history of either a genetic disorder or hereditary syndrome (88.4%, *n* = 267) or cancer (53.3%, *n* = 161). Only 33.1% (*n* = 100) were involved in genetics or genomics within their professional activities. Information on genetic testing has been addressed during both undergraduate (57.3%, *n* = 173) and postgraduate training (48%, *n* = 145). The main areas of professional activity were statistics and epidemiology (36%) and PH policy (33.4%).

#### Knowledge on DTC-GT

The majority of the respondents were aware about DTC-GTs’ web purchase (74.5%, *n* = 225) and that companies are selling these tests (78.5%, *n* = 237), reporting Internet (53.3%, *n* = 161) and medical journal articles (40.4%, *n* = 122) as the main sources of information. Only 48 individuals (15.9%) had ever heard about professional organizations with statements or recommendations regarding DTC-GTs, mainly referring FDA (*n* = 13) and European Society of Human Genetics (*n* = 7), whereas 8.3% (*n* = 25) were aware about any national legal framework related to DTC-GT.

As for the clinical application of DTC-GTs, more than half were aware for the following applications: hereditary breast (72.2%, *n* = 218) and ovarian cancer (57.3%, *n* = 173), ancestry (61.3%, *n* = 185) and paternity (58.6%, *n* = 177) (table 2).

**Table 2:** Respondent’s awareness and knowledge on DTC-GTs

Awareness and knowledge	*N*	%
Awareness that companies are advertising and selling DTC-GTs	237	78.48
Source of information		
Internet	161	53.31
Journal articles/medical journals	122	40.40
Patients colleagues	76	25.17
Medical websites	68	22.52
Scientific meetings	60	19.87
Magazines and newspapers	58	19.21
TV/radio	41	13.58
Professional societies	39	12.91
Selling companies	23	7.62
Government agencies	12	3.97
Awareness that DTC-GTs can be purchased on the web	225	74.5
Awareness about DTC-GTs selling companies		
None	136	45.03
23andMe	122	40.40
Ancestry	84	27.81
deCODEme	56	18.54
Navigenics	24	7.95
My heritage	10	3.31
Other (DanteLabs, OncoDNA, TellmeGen.…)	7	2.32
Awareness of professional organizations that have issued a position statement/opinion/recommendation regarding DTC-GTs		
Yes	48	15.89
No	166	54.97
I don’t know	88	29.14
Awareness about a current satisfactory legal framework in your country that covers aspects particularly related to DTC-GTs		
Yes	25	8.28
No	74	24.5
I don’t know	203	67.22
Knowledge about the EU countries has implemented national legislation on genetic testing that can affect the provision of DTC-GTs		
I don’t know	234	77.48
Germany	44	14.57
France	35	11.59
Belgium	24	7.95
UK	22	7.28
Italy	21	6.95
Netherlands	15	4.97
Austria	8	2.65
Spain	8	2.65
Sweden	8	2.65
Knowing about additional protocol to the convention on human rights and biomedicine, concerning genetic testing for health purposes		
Yes	91	30.13
No	143	47.35
I don’t know	68	22.52
Knowledge of DTC-GTs application		
Testing for hereditary breast cancer	218	72.19
Ancestral tests	185	61.26
Paternity testing	177	58.61
Testing for hereditary ovarian cancer	173	57.28
Testing for Alzheimer disease	120	39.74
Testing for Type 2 diabetes	104	34.44
Testing for hereditary Mendelian disorders	125	41.39
Testing for familial hypercholesterolemia	106	35.10
Nutrigenomics testing	109	36.09
Testing for lynch syndrome (hereditary non-polyposis colorectal cancer)	111	36.75
Pharmacogenomics testing	111	36.75
Testing for prostate cancer	82	27.15
Testing for depression	79	26.16
Testing for lung cancer	75	24.83
Testing for skin cancer	70	23.18
Testing for acute myeloid leukaemia	68	22.52
Athletic ability	56	18.54
Child talent	38	12.58
Infidelity	36	11.92

#### Attitudes towards DTC-GTs

The majority of the respondents had negative attitude (32.1%, *n* = 97 strongly disagree, 29.1%, *n* = 88 disagree) regarding the provision of DTC-GTs without an established physician–patient relationship and without a face-to-face consultation, and positive attitudes (47.4%, *n* = 143 strongly agree; 31.8%, *n* = 96 agree) regarding the use of these tests only after demonstration of clinical validity and utility. Almost 34.4% (*n* = 104) considered DTC-GTs currently clinically useful. Nearly half (35.1%, *n* = 106 agree; 19.2%, *n* = 58 strongly agree) agreed that DTC-GTs should be introduced only if economic evaluations show favourable cost-effectiveness ratios compared with alternative health interventions (Supplementary material S3).

DTC-GTs’ results were reported to be both helpful and harmful in 59.6% (*n* = 180). The provision of DTC-GTs should be regulated on a national level for 70.5% (*n* = 213) and should include a qualified health professional (91.4%, *n* = 276), only 16.9% (*n* = 51) were feeling prepared to answer citizen’s questions (table 3).

**Table 3:** Respondents’ attitudes towards DTC-GTs

Attitudes	*N*	%
Overall, do you feel that the results of DTC-GT can be helpful or harmful for individual’s health decisions?		
Both helpful and harmful	180	59.6
I don’t know	48	15.89
Harmful	38	12.58
Helpful	36	11.92
Should a qualified health professional be involved in the DTC-GT process?	276	91.39
Who would be the most appropriate to provide counselling to an individual following a DTC-GT?		
Genetic specialist	179	59.27
General practitioner	47	15.56
I don’t know	43	14.24
Both GPs and genetic specialists	10	3.31
Company providing the test	10	3.31
Other	13	4.3
Do you feel prepared to answer a citizen’s questions about DTC-GT?		
Yes	51	16.89
No	198	65.56
I don’t know	53	17.55
Should the provision of DTC-GT be regulated on a national level in a similar way as medicines?		
Yes	213	70.53
No	17	5.63
I don’t know	72	23.84
If yes, which aspects of DTC-GT should be regulated?		
Evidence of clinical validity of DTC-GT	187	61.9
Accreditation of the laboratory producing DTC-GT	174	57.6
Genetic counselling	171	56.6
Informed consent process	168	55.6
Provision of medical supervision	155	51.3
Storage of and access to results	152	50.3
Advertising of DTC-GT	130	43
Access to tests	110	36.4

Considering the potential benefits of DTC-GTs (Supplementary material S4), more than half of the respondents had positive attitude towards the enabling people to learn about their genetic conditions at risk (48%, *n* = 145 agree; 18.5%, *n* = 56 strongly agree), while regarding the potential risks of DTC-GTs (Supplementary material S5), most of the respondents considered questionable the analytical validity (38.4%, *n* = 116 agree; 18.5%, *n* = 56 strongly agree) and clinical utility (41.4%, *n* = 125 agree; 22.5%, *n* = 68 strongly agree).

#### Behaviours towards DTC-GTs

Around 94% (*n* = 284) never underwent a DTC-GT, and among them only 34.6% (*n* = 100) would not undergo these tests personally in the future. More than one-third (*n* = 109) never visited a website offering DTC-GTs and 15 respondents (4.97%) never referred a citizen to these websites. One-quarter was asked about DTC-GT by citizens during the previous year, mainly to know about the test in general (60.6%) and the benefits of testing (42.3%), and 95% (*n* = 287) had never encouraged a citizen to undergo the tests (table 4).

**Table 4:** Respondents’ behaviours towards DTC-GTs

Behaviours	*N*	%
Ever performed a DTC-GT	18	5.96
If no, willingness to personally undergo a DTC-GT		
Yes	88	30.45
No	100	34.60
Don’t know	101	34.95
Ever visited a website offering DTC-GT	109	36.09
Ever encouraged a citizen to undergo a DTC-GT	25	8.28
In the past years, have received question about DTC-GT by a citizen	76	25.17
If yes, in the past year, how many citizens asked questions about DTC-GTs for cancer risk prediction		
<2	100	77.52
>2	29	22.48
If yes, in which of the following categories can the citizens’ question be included		
Knowledge about the test(s)	63	60.58
The benefits of testing	44	42.31
Impact on patient’s care	26	25
Knowledge about the company/companies that is/are offering the test	23	22.12
The appropriateness of the test cost related to the type of information they will obtain	18	17.31
Ever referred a citizen to a specific website offering DTC-GT	15	4.97

#### Multivariable analysis

Supplementary tables S6–S8 summarize the results of the multivariable logistic regression analysis.

Predictors of knowledge about DTC-GTs were the involvement in genetics/genomics and receiving training during under- or post-graduate studies [adjusted OR 2.90, 95% CI (1.70–4.95), *P* < 0.0001; and adjusted OR 2.09, 95% CI (1.02–4.30), *P* = 0.043, respectively] (Supplementary table S6).

A positive attitudes towards DTC-GTs was significantly associated with medical degree and knowledge about DTC-GTs [adjusted OR 2.07, 95% CI (1.23–3.50), *P* = 0.006; and adjusted OR 1.82, 95% CI (1.07–3.20), *P* = 0.027, respectively]. In addition, a borderline inverse association was observed between age and attitudes towards DTC-GTs [adjusted OR 0.97, 95% CI (0.95–1.00), *P* = 0.006] (Supplementary table S7).

None of the selected covariates was predictive of a favourable behaviour of healthcare professionals towards DTC-GTs (Supplementary table S8).

## Discussion

Our survey on knowledge, behaviours and attitudes towards DTC-GTs of PH professionals affiliated to EUPHA revealed high level of awareness on DTC-GTs being commercialized and purchasable on the web. Although aware of DTC- GT, most of the participants did not feel prepared to answer citizens’ questions. The awareness mostly on oncological applications of DTC-GT is in line with the results of the survey at a EU level on genomics knowledge among PH professionals.^3^ Attitudes regarding the provision of DTC-GTs without the provision of a healthcare professional were negative and nearly all the respondents agreed for the involvement of a qualified health professional in the DTC-GT process.

Our findings show an important overview on the main policy issues related to DTC-GTs, reflecting the presence of a fragmented regulatory framework and scarce awareness of regulatory provisions.^26^ The positive attitudes towards the introduction of DTC-GTs in the healthcare system after the proven clinical effectiveness support the European consensual points to be considered for prioritizing clinical genetic testing services.^27^

Despite the provision in most of the participants of genetics/genomics information during university and further studies for most of the participants, our results underline that DTC-GTs are still a new reality. In fact, from our analysis, the involvement in genetics/genomics in the professional activities and the training during under- and post-graduate studies were associated with higher awareness and knowledge on the matter. However, the willingness to undergo DTC-GTs, and, more generally, a favourable behaviour towards DTC-GT, was not associated with the healthcare professionals’ background. That might be because the received information during training, or the knowledge deriving from a positive history of cancer or hereditary disorders, was general and did not cover all the aspects of genetic testing. Hence, there is the need to reformulate and update the training courses of healthcare professionals and to provide continuum education in line with technological developments, with particular regard to personalized medicine and implementation in clinical practice. Also, information about genetic tests should be provided to patients and citizens in the most comprehensive way, to empower them in making informed choices. Since behaviour does not seem to be influenced by the education and training of health professionals, nor by the other variables considered, it might be useful to identify some reasons, through further research on this evidence. In addition, health professionals and citizens should be evaluated in parallel, in fact, considering the aspects that modify people’s behaviour towards DTC-GTs could help to understand how to evaluate the behaviour of health professionals. This aspect could be addressed in a complementary way by educational programmes, providing practical suggestions to healthcare professionals together with notions and guidelines.

Respondents reported to be unfavourable to the supply of DTC-GT without an established doctor–patient relationship and without a face-to-face consultation, similar to the majority of European geneticists.^22^ The proportion of 17% of professionals, who felt prepared for the DTC-GT results’ interpretation, is in accordance with previous studies, reporting 16% of primary care physicians,^28^ 5% of general physicians^21^ or 7% of genetic specialists and counsellor^15^ feeling knowledgeable enough and confident regarding the results interpretation.

This low percentage, coupled with the fact that only 16% of them had information on professional organizations with statements or recommendations related to DTC-GT, underlines the need for a targeted improvement of curricula, especially in the training of GPs. Furthermore, some remarks could be made about the synergetic role of GPs and PH professionals. They are at the forefront of managing the needs of patients/citizens and PH care, which is deemed necessary for DTC-GTs. According to a recent survey, GPs have a significant training gap and DTC-GTs could influence their daily practices.^29^*Ad hoc* tools could therefore be designed, in synergy with PH professionals and their training and representative bodies (e.g. Association of Schools of Public Health in the European Region—ASPHER, EUPHA), who should become ambassadors of information and education, both towards their medical colleagues (and health professionals) and the general public.

However, our results confirm the conclusions drawn by a previously published systematic review on knowledge and opinions of health professionals towards DTC-GTs that reported a very low level of preparation and little confidence in interpreting DTC-GT results.^7^ Similar to this systematic review, the attitudes about the clinical usefulness of DTC-GTs were controversial by the majority of the participants, considered these tests as both useful and harmful. PH professionals had positive attitudes on the potential benefits and concerns towards the perceived risks, mainly about the clinical validity and data privacy and confidentiality. The confidentiality of information has been perceived as a benefit of DTC-GTs by GPs, who are reported to perceive more benefits of DTC-GTs than genetic counsellors.^23^ PH professionals participating in our survey had little experience with patients asking about DTC-GTs or discussing testing results with them, similar to other healthcare professionals, such as genetic specialist and genetic counsellors^15^^,^^22^ or primary care physicians.^17^

In our survey, the response rate was low, approximately <10% of the EUPHA members. However, this response rate is in line with a former survey,^3^ and also with other studies using the web-survey method, where the response rate varies from 6% to 15%.^30^ According to several systematic reviews and meta-analyses, the nature of the topic is one of the most important factors that influence response rates in web-based surveys.^31^^,^^32^ The obtained response rate was not unexpected considering the specific topic evaluated, which is not particularly common compared to other health technologies. Results of a study among GPs and clinical physicians, reported that response rates of web-surveys vary by healthcare profession specialist.^33^ Another factor influencing the response rate is the number of reminders.^32^ There is inconsistency regarding the timing and the frequency of follow-up reminders,^34^ even though it has been shown that one follow-up reminder may increase the response rates,^35^ whereas too many may be considered as a harassment to the respondents.^36^ The web-based format did not allow us also to explore and address the bias associated with non-response. A literature review reported that responding and non-responding physicians tend to share similar characteristics,^37^ but in the present study, it was not possible to compare respondents and non-respondents. Another limitation of the web-based surveys is that constrains the participation of older individuals, who are more comfortable with paper rather than internet-based questionnaire or have limited Internet access.^38^ The mean age of the interviewees underlines the greater interest of young people in the participation and contribution to research projects. The female predominance is in accordance with previous studies reporting that women physicians are more likely to participate in surveys.^32^

Lastly, the survey was not validated through a pilot test; however, it was developed on the basis of the literature review, incorporated feedback from PH experts and geneticists, gathered through the focus group validation process.

To our knowledge, this is the first survey at the European level, including not only the EU MS, on the knowledge, attitudes and behaviour of professionals in PH. Few studies have been conducted in Europe regarding healthcare professionals’ perspectives towards DTC-GTs, two at the European level,^22^^,^^39^ one in Greece^18^ and the other one in Italy.^21^ However, none of these studies investigated PH professionals, focusing on clinical geneticists,^22^^,^^27^ GP^21^ and physicians.^18^

The sample of respondents is, by composition, representative of EUPHA members, since the majority consists of health professionals working in the academic or research sectors.^3^ An important strength of our survey is the methodological part, since it has been carefully structured on the basis of a review of the literature and a focus group validation by experts in the field of PH, genetics and genomics in PH. Therefore, this survey could be used by other researchers to develop similar studies at the national level and beyond, in order to inform decision-makers about the state of the art of the PH professionals’ literacy on DTC-GT in their countries.

To conclude, the majority of PH professionals reported a high level of awareness on DTC-GTs web purchase and moderate to low level of awareness towards their applications. PH professionals agreed that a qualified health professional should be involved in the DTC-GT process and considered questionable the tests’ analytical validity and clinical utility. Given that the majority of PH professionals did not feel prepared to answer citizens’ questions regarding DTC-GTs, training and education initiatives are needed at the European level, updated in line with genomics advancements. The results of the iPAAC Joint Action and the oncogenomics course developed within it represent the starting point for an integrated update of training programmes for European PH professionals on this topic.^40^ New joint actions and further EU-funded projects should continue the iPAAC legacy, recognizing the increasing use of DTC genetic testing by the general population. Moreover, since the training of health professionals is vital for ensuring the quality of care delivery and PH advocacy, it is clear that this aspect, together with literacy and citizen empowerment, must be integrated into any health research project and initiative. In addition, potential proposed solutions, in terms of training and educational programs, should be evaluated for their effectiveness and sustainability, measuring the impact on health professionals’ knowledge, attitudes and behaviours in the medium and long term.

## Supplementary data

Supplementary data are available at *EURPUB* online.

## Supplementary Material

ckac139_Supplementary_Data
