# Supplementary material for: Survey of Professionals of the European Public Health Association (EUPHA) towards Direct-to-Consumer Genetic Testing
Source: Eur J Public Health. 2022 Sep 30;33(1):139–45. doi: 10.1093/eurpub/ckac139 (PMC9898004; doi:10.1093/eurpub/ckac139)
Supplement: ckac139_Supplementary_Data [file ckac139_supplementary_data.docx]

**S1.** Characteristics and main results of the eleven studies included in the literature review of surveys on DTC-GTs.

| **Author, year [ref]** | **Country** | **Number of participants;**  **Male (%)** | **Category of participants** | **Knowledge** | **Attitudes** | **Behaviors** |
| --- | --- | --- | --- | --- | --- | --- |
| Goddard KA, 2007 [19] | USA | 1,250;  843 (67.4%) | GPs and pediatricians | -44% of physicians were aware of DTCngts  -Main sources of information: 62% media (television, radio or newspaper) | Not applicable | - 76% of the physicians aware of DTCngts, reported that <1% of their patients had asked about such tests  - 93% reported that <1% of their patients had discussed results of a DTCngt with them. |
| Ohata T, 2009 [23] | Japan | 1,399;  1,159 (82.8%) | GPs and CGs | - aware of DTC-GTs: 38.0% of GPs and 68.4% of CGs  -Main sources of information: Internet, TV/newspapers/magazines, and scientific meetings/journals | - GPs perceived more benefits of DTC-GTs compared with CGs, CGs focused more on the risks.  - Benefits considered: convenience (allowing consumers staying far from medical institutions to undergo tests) and confidentiality of information.  - Concerning risks: understanding of results and advertising. | Not applicable |
| Giovanni MA, 2010 [25] | USA | 133 | Genetic specialists and GCs | Not applicable | 52.3% deemed DTC-GTs to be clinically useful. | - 73.3% reported only one referral was made to other providers based on testing results,  - referrals to geneticists or genetic counselors (35.3%) or breast care specialists or surgeons (23.5%) |
| Hock KT, 2011 [24] | USA | 312 | GCs | - 40% correct responses on which diseases can be tested DTC  - 54% correct responses about regulation and oversight  - 37% correct responses on which organizations have issued a position statement | - genetic counselors have a professional obligation to be knowledgeable about DTC-GTs (55%) and interpret results (48%).  - 51% of respondents thought genetic testing should be limited to a clinical setting.  - 56% agreed direct-to-consumer genetic testing is acceptable if genetic counseling is provided;  - 70% would consider DTC-GTs for patients with concerns about genetic discrimination, geographic constraints or anonymity requests | - 75% had visited a DTC-GT website  - 15% and 14% had suggested DTC-GTs as an option for a patient or received a request to interpret DTC-GT results for a patient, respectively  - 11% had referred a patient to a specific DTC-GT website, while 8% told a patient to search online for DTC-GTs. |
| Mai Y, 2011 [18] | Greece | 496;  (48.3%) | Physicians from all medical specialties | 11.9% believe that there is a satisfactory legal framework in Greece to cover aspects of genetic testing | - 12.7% in favor of direct-access genetic testing  - 89.7% of those against believed that a physician should refer individuals to a genetic testing laboratory, only 5% believed that a pharmacist should be allowed to do this | -48.5% had encouraged their patients to undergo genetic testing  -74.5% would undergo genetic testing |
| Brett GR, 2012 [15] | Australia | 168 | Genetic specialists and GCs | 7% of respondents were confident in accurately interpreting and explaining DTC-GT results | Most do not consider DTC-GT useful for individuals:   - who want anonymous testing (54%) - are driven by curiosity (54%) - are geographically isolated (60%)   48% consider DTC-GT useful for individuals who are provided appropriate genetic counselling  Only 8% deemed DTC-GTs clinically useful in their practice | 11% of respondents reported having had one or more clients consult with them after undertaking DTC-GT  61% would not consider undergoing DTC-GT themselves |
| Powell KP, 2012 [17] | USA | 382;  263 (69.6%) | PCPs (family physicians and internists) | 61.3% had never heard or read about DTC-GTs, 38.7% were aware of DTC-GT existence  Most common sources of information: medical or scientific journals (35.1%), television (33.1%), newspaper articles (28.4%) and the Internet (27.0%)  85% did not feel prepared to answer their patient’s questions regarding DTC-GTs | 42.6% of the respondents who were aware of DTC-GTs thought that testing was clinically useful  The most frequently benefits were considered the ability to:   - offer screening tests at an earlier age to individuals at an increased risk (82.5%), - offer screening tests more frequently to individuals with an increased risk (81.0%)   The most common concerns were: results could increase patient anxiety (87.1%), patients may interpret the results incorrectly (85.1%), advertisements may mislead patients (85.1%), and the clinical utility is questionable (81.8%) | A majority of the PCPs who were aware of DTC-GTs (81.1%) had never discussed tests with a patient or had a patient bring in results of DTC-GTs  Four of the five PCPs who had patients bring in results reported they did not change their patient’s medical management, while the remaining physician recommended lifestyle changes including changes in diet and supplements |
| Ram S, 2012 [20] | New Zealand | 113;  64 (56.6%) | GPs | 47.8% of respondents had heard about DTC-GTs | Respondents considered convenience to be the greatest benefit for the individual requesting DTC-GT (64.6%).  Misunderstanding of results (92.2%) and inadequate provision of information (91.2%) were perceived to be the greatest risks associated  Lack of knowledge, experience and time were all considered barriers to GPs providing genetic counselling, and a genetic specialist was highlighted as the most appropriate to provide it by 61.1% of respondents |  |
| Howard HC, 2013 [22] | 28 European Countries | 131;  66 (50.4%) | CGs | 86% of respondents were aware that companies are advertising and selling genetic tests directly to consumers | 84% of respondents disagreed with replacing face-to-face medical supervision by a medical doctor with telephone supervision outside of the context of an established doctor-patient relationship | 34% of the respondents have been contacted by at least one patient who addressed the DTC-GT subject but had not (yet) undergone a DTC-GT  44% of the respondents have had at least one patient contact them after having undergone a DTC-GT |
| Mainous AG, 2013 [16] | USA and Canada | 1,311;  (55.0%) | Family physicians | 54.4% of respondents felt that they had poor knowledge about genetic tests | 71.8% felt that genetic testing was valuable for the purpose of determining a patient’s risk for disease in primary care, while 56% reported it as valuable for determining the best treatment for a patient  58.1% agreed that DTC-GTs are more likely to harm a patient’s general health decisions, while 10.3% felt they are more likely to help | 70.5% of the respondents reported they were never asked about DTC-GTs by patients |
| Baroncini A, 2015 [21] | Italy | 114;  51 (44.7%) | GPs | 68.4% were not aware that companies are selling genomic tests directly to consumers, 31.6% were aware  95.6% felt unprepared to answer patients’ questions on DTC-GT  Main sources of information: TV/radio (20.5%), medical journals (15.4%), and magazines/newspapers (14.1%) | 61.1% of the aware respondents deemed the DTC-GT for chronic complex diseases to be not  clinically useful | 30.6% of the aware respondents had already been approached by patients about DTC-GT |

*Abbreviations*: GP: general practitioner; CG: clinical geneticist; GC: genetic counselor; DTCngts: direct-to-consumer nutrigenomic tests; DTC-GT: direct-to-consumer genetic test; PCP: primary care physician


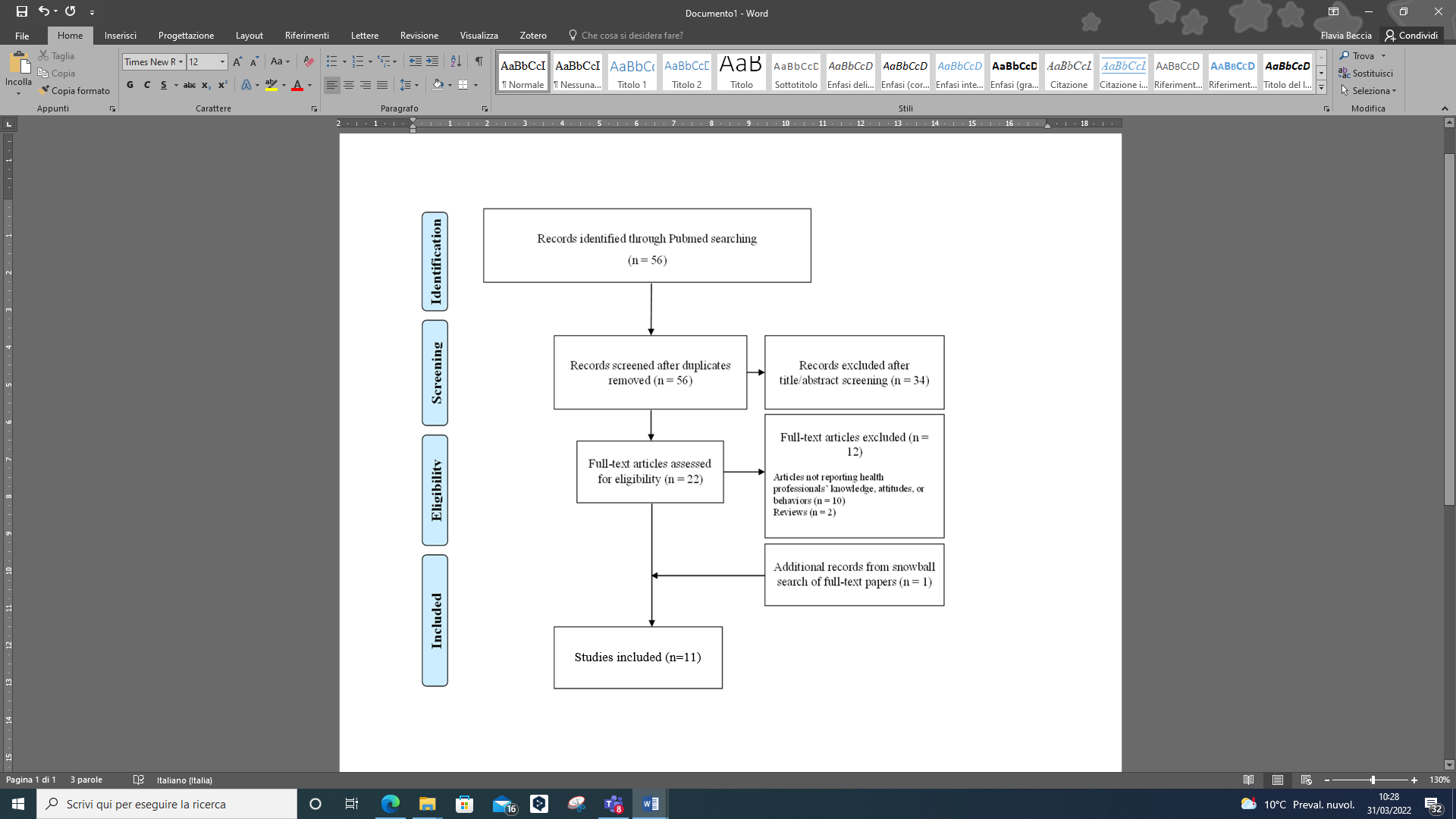


**Figure S1.** PRISMA flow diagram of the study screening and selection process.

**S2**. Questionnaire on Knowledge, Attitudes and Behaviors about Direct-To-Consumer Genetic Tests

**PERSONAL DETAILS**

*In this section you are asked to provide some personal details.*

**Birth Year** __________________________________

**Gender**

- F
- M

**In which Country do you conduct your professional activity?**
**Do you have a personal or family history of a genetic disorder or hereditary syndrome?**

- Yes
- No

**Do you have a personal or family history of cancer?**

- Yes
- No

**PROFESSIONAL ACTIVITY**

*In this section you are asked to provide some information on your education and your professional activities.*

**Are you involved in genetic/genomics within your professional activities?**

- Yes
- No

**What is the highest educational degree obtained?**

- Bachelor‘s
- Master’s
- Doctorate
- Other (please specify): _________________

**Area of degree**

- Medicine
- Health professions (nursing, etc.)
- Biology
- Psychology
- Statistics
- Economics
- Other or mixes (please specify) _________________________

**Has information on genetic testing been addressed during your undergraduate training?**

- Yes
- No

**Has information on genetic testing been addressed during your postgraduate training?**

- Yes
- No
- Not applicable

**Which sector do you work in?**

- Academic/research
- Hospital
- Technical Agency
- National Government (i.e. Ministry of Health)
- Local Government (i.e. Regional Health Authorities)
- Public health service (e.g. vaccination service/screening program/maternal-child health service, etc.)
- Other (please specify): _________________

**Which are your main areas of work? (maximum 3 answers are allowed)**

- Statistics and epidemiology
- Communicable diseases control
- Non-communicable diseases control
- Cancer prevention
- Environment related diseases control
- Health technology assessment
- Health impact assessment
- Health services research
- Health services management
- Public health policy
- Health economics
- Migrant and ethnic minorities health
- Food and nutrition
- Public mental health
- Public health genomics
- Injury prevention and safety promotion
- Child and adolescent health
- Others (please specify): _________________

**KNOWLEDGE**

*In this section you are asked about your knowledge on DTC GT*

**Are you aware that companies are advertising and selling genetic tests directly to consumers** (DTC GTs)**?**

- Yes
- No
- I don’t know

**Where have you heard or read about DTC GTs?**

- Journal articles/Medical Journals
- Government agencies
- Medical Web sites
- Tv/Radio
- Professional Societies
- Patients, Colleagues
- Selling Companies
- Internet
- Magazines and newspapers
- Scientific Meetings
- Other (please specify): _________________

**Are you aware that DTC GTs can be purchased on the Web?**

- Yes
- No
- I don’t know

**Which of the following companies selling these tests have you heard about?**

- 23andMe
- deCODEme
- Navigenics
- Ancestry
- Other (please specify): _________________

**DTC companies offer variety of services, either health or non-health related. Are you familiar with the following applications of DTC GTs? - *Yes/No/I don’t know***

- Testing for hereditary breast cancer
- Testing for hereditary ovarian cancer
- Testing for Lynch syndrome (hereditary nonpolyposis colorectal cancer)
- Testing for lung cancer
- Testing for Acute myeloid leukemia
- Testing for Prostate cancer
- Testing for Skin cancer
- Testing for Familial hypercholesterolemia
- Testing for Type 2 diabetes
- Testing for Alzheimer disease
- Testing for Depression
- Paternity testing
- Ancestral tests
- Testing for hereditary Mendelian disorders
- Nutrigenomics testing
- Pharmacogenomics testing
- Athletic ability
- Child talent
- Infidelity

**Have you heard of professional organizations that have issued a position statement / opinion / recommendation regarding DTC GTs?**

- **Yes**
- **No**
- **I don’t know**

**If yes, could you please indicate at least one of these organizations?** _________________

**Is there currently a satisfactory legal framework in your country that covers aspects particularly related to DTC GTs?**

- Yes
- No
- I don’t know

**As far as you know, which EU countries has implemented national legislation on genetic testing that can affect the provision of DTC GTs?**

- Austria
- Belgium
- Bulgaria
- Croatia
- Cyprus
- Czechia
- Denmark
- Estonia
- Finland
- France
- Germany
- Greece
- Hungary
- Ireland
- Italy
- Latvia
- Lithuania
- Luxembourg
- Malta
- Netherlands
- Poland
- Portugal
- Romania
- Slovakia
- Slovenia
- Spain
- Sweden
- United Kingdom
- I don’t know

**Have you heard about Additional Protocol to the Convention on Human Rights and Biomedicine,**

**concerning Genetic Testing for Health Purposes?**

- Yes
- No

**ATTITUDES ON DTC GT**

*In this section you are asked about your opinion regarding DTC GTs.*

**How do you feel about the provision of different types of GTs outside of the traditional healthcare setting, without an established physician-patient relationship and without a face-to-face consultation?**

- Strongly disagree
- Somewhat disagree
- Neither agree or disagree
- Somewhat agree
- Strongly agree

**Do you think information regarding DTC GTs are easily accessible to physicians?**

- Yes
- No
- I don’t know

**Please express your opinion on the following two sentences:**

**“DTC GTs that can identify an increased risk of developing a disease, including cancer, should be introduced only after demonstration of clinical validity and utility.”**

- Strongly agree
- Agree
- Neither agree nor disagree
- Disagree
- Strongly disagree

**“DTC GTs that can identify an increased risk of developing a disease, including cancer, should be introduced only if economic evaluations show favourable cost-effectiveness ratios compared with alternative health interventions.”**

- Strongly agree
- Agree
- Neither agree nor disagree
- Disagree
- Strongly disagree

**To what extent do you agree with the following potential benefits of DTC GTs?**

**(Strongly agree/Agree/neither agree nor disagree/ disagree/strongly disagree)**

- Provision of useful service in delivery of healthcare
- Consumers staying far from medical institutions can undergo tests
- The promotion of preventive medicine by using convenient genetic tests can be expected
- Personalized services according to the genetic makeup of each individual can be provided
- Consumers can undergo tests in private
- Raising awareness about genetics
- Individuals may learn about genetic conditions they may be at risk for
- Encouraging individuals to take responsibility for their health
- Stimulating individuals to facilitate family discussions surrounding their personal health
- Positive impact on the patient-doctor relationship

**To what extent do you agree with the following potential risks of DTC GTs?**

**(Strongly agree/Agree/Neither agree nor disagree/ Disagree/Strongly disagree)**

- The analytical validity, or accuracy, of the test results is questionable
- The clinical utility, or ability to use the results in practice, is questionable
- Lack of provision of adequate information and adequate counseling after the test
- Advertisements may mislead patients
- There is a possibility that the results will lead to discrimination in employment and insurance
- There is a possibility that beliefs such as genetic determinism (i.e. the belief that human physical and mental well-being are genetically pre-programmed and cannot be influenced by behavioral, environmental and social factors) will spread
- Genetic information may not be kept confidential by the DTC GT companies
- Patients may interpret the results incorrectly
- Results could increase patient anxiety
- Gaining a false sense of security from a negative test result
- Encouraging individuals to get unnecessary genetic tests carried out
- Encouraging individuals to get unwarranted procedures done based on the results of the DTC GT
- Puts a strain on health resources that could be more effectively utilized
- Negative impact the patient-doctor relationship
- Physicians may feel obligated to refer patients to specialists, perhaps unnecessarily
- Physicians may feel obligated to refer patients for follow-up procedures, perhaps unnecessarily

**Overall, do you feel that the results of DTC GT can be helpful or harmful for individual’s health decisions?**

- helpful
- harmful
- both helpful and harmful
- I don’t know

**In general, do you think DTC GTs are currently clinically useful?**

*(meaning that individual’s test results should be taken into consideration when formulating a medical management plan (e.g. when to refer for screening tests, when to refer to a specialist, etc.))?*

- Strongly agree
- Agree
- Neither agree nor disagree
- Disagree
- Strongly disagree

**Should a qualified health professional be involved in the DTC GT process?**

- Yes
- No

**Who would be the most appropriate to provide counselling to an individual following a DTC GT?**

- Genetic specialist
- Company providing the test
- General Practitioner
- I don't know
- Others (please specify): _________________

**Do you feel prepared to answer a citizen’s questions about DTC genetic testing?**

- Yes
- No
- I don’t know

**Should the provision of DTC GT be regulated on a national level in a similar way as medicines?**

- Yes
- No
- I don’t know

**If yes, which aspects of DTC GT should be regulated?**

- Accreditation of the laboratory producing DTC GT
- Evidence of clinical validity of DTC GT
- Advertising of DTC GT
- Informed consent process
- Provision of medical supervision
- Genetic counselling
- Storage of and access to results
- Access to tests

**If yes, which institution or authority should provide regulation in your country? ___________**

**BEHAVIORS**

*In this section we will ask you about your personal behavior regarding DTC GTs*

**Have you ever undergone a DTC GT?**

- Yes
- No

**If no, would you be willing to personally undergo a DTC GT?**

- Yes
- No
- Don’t know

**Have you ever visited a website offering DTC GT?**

- Yes
- No

**Have you ever encouraged a citizen to undergo a DTC GT?**

- Yes
- No

**In the past years, have you ever been asked about DTC GT by a citizen?**

- Yes
- No

**If yes, in the past year, how many citizens asked questions about DTC GTs for cancer risk prediction?**

- ≤2
- >2

**If yes, in which of the following categories can the citizens’ question be included?**

- Knowledge about the company/companies that is/are offering the test
- Knowledge about the test(s)
- The benefits of testing
- Impact on patient’s care
- The appropriateness of the test cost related to the type of information they will obtain
- Other (please specify): _________________

**Have you ever referred a citizen to a specific website offering DTC GT?**

- Yes
- No

**Table S3**. Respondents’ attitudes towards the statements formulated on DTC-GTs.

|  | **Strongly disagree** | **Disagree** | **Neither agree or disagree** | **Agree** | **Strongly agree** |
| --- | --- | --- | --- | --- | --- |
| **Attitudes** | **N (%)** | **N (%)** | **N (%)** | **N (%)** | **N (%)** |
| *Provision of different types of GTs outside of the traditional healthcare setting* | 97 (32.12) | 88 (29.14) | 74 (24.50) | 38 (12.58) | 5 (1.66) |
| *Health related DTC-GTs should be introduced only after demonstration of clinical validity and utility* | 2 (0.66) | 12 (3.97) | 49 (16.23) | 96 (31.79) | 143 (47.35) |
| *Health related DTC-GTs should be introduced only if economic evaluations show favourable cost-effectiveness ratios compared with alternative health interventions* | 9 (2.98) | 42 (13.91) | 87 (28.81) | 106 (35.10) | 58 (19.21) |
| *DTC-GTs are currently clinically useful* | 16 (5.30) | 55 (18.21) | 127 (42.05) | 92 (30.46) | 12 (3.97) |

**Table S4.** Respondents’ attitudes on potential benefits of DTC-GTs

|  | **Strongly disagree** | **Disagree** | **Neither agree or disagree** | **Agree** | **Strongly agree** |
| --- | --- | --- | --- | --- | --- |
| **Attitudes** | **N (%)** | **N (%)** | **N (%)** | **N (%)** | **N (%)** |
| *The analytical validity, or accuracy, of the test results is questionable* | 0 (0.00) | 24 (7.95) | 106 (35.10) | 116 (38.41) | 56 (18.54) |
| *The clinical utility, or ability to use the results in practice, is questionable* | 2 (0.66) | 28 (9.27) | 79 (26.16) | 125 (41.39) | 69 (22.85) |
| *Lack of provision of adequate information and adequate counseling after the test* | 0 (0.00) | 7 (2.32) | 66 (21.85) | 120 (39.74) | 109 (36.09) |
| *Advertisements may mislead patients* | 0 (0.00) | 12 (3.97) | 53 (17.55) | 115 (38.08) | 122 (40.40) |
| *The results could lead to discrimination in employment and insurance* | 7 (2.32) | 20 (6.62) | 80 (26.49) | 114 (37.75) | 81 (26.82) |
| *There is a possibility that beliefs such as genetic determinism will spread* | 8 (2.65) | 28 (9.27) | 88 (29.14) | 114 (37.75) | 64 (21.19) |
| *Genetic information may not be kept confidential by the DTC-GT companies* | 7 (2.32) | 22 (7.28) | 69 (22.85) | 107 (35.43) | 97 (32.12) |
| *Patients may interpret the results incorrectly* | 0 (0.00) | 10 (3.31) | 37 (12.25) | 101 (33.44) | 154 (50.99) |
| *Results could increase patient anxiety* | 0 (0.00) | 14 (4.64) | 35 (11.59) | 119 (39.40) | 134 (44.37) |
| *Gaining a false sense of security from a negative test result* | 0 (0.00) | 12 (3.97) | 58 (19.21) | 122 (40.40) | 110 (36.42) |
| *Encouraging individuals to get unnecessary genetic tests carried out* | 3 (0.99) | 25 (8.28) | 66 (21.85) | 108 (35.76) | 100 (33.11) |
| *Encouraging individuals to get unwarranted procedures done based on the results of the DTC-GT* | 4 (1.32) | 22 (7.28) | 67 (22.19) | 115 (38.08) | 94 (31.13) |
| *Putting a strain on health resources that could be more effectively utilized* | 4 (1.32) | 25 (8.28) | 99 (32.78) | 107 (35.43) | 67 (22.19) |
| *Negative impact the patient-doctor relationship* | 4 (1.32) | 57 (18.87) | 110 (36.42) | 89 (29.47) | 42 (13.91) |
| *Physicians may feel obligated to refer patients to specialists, perhaps unnecessarily* | 3 (0.99) | 41 (13.58) | 91 (30.13) | 114 (37.75) | 53 (17.55) |
| *Physicians may feel obligated to refer patients for follow-up procedures* | 3 (0.99) | 43 (14.24) | 85 (28.15) | 114 (37.75) | 57 (18.87) |

**Table S5.** Respondents’ attitudes on potential risks of DTC-GTs

|  | **Strongly disagree** | **Disagree** | **Neither agree or disagree** | **Agree** | **Strongly agree** |
| --- | --- | --- | --- | --- | --- |
| **Attitudes** | **N (%)** | **N (%)** | **N (%)** | **N (%)** | **N (%)** |
| *Provision of useful service in delivery of healthcare* | 14 (4.64) | 25 (8.28) | 80 (26.49) | 40 (13.25) | 143 (47.35) |
| *Consumers that live away from medical institutions can undergo tests* | 30 (9.93) | 71 (23.51) | 91 (30.13) | 90 (29.80) | 20 (6.62) |
| *The promotion of preventive medicine by using convenient genetic tests* | 11 (3.64) | 37 (12.25) | 78 (25.83) | 133 (44.04) | 43 (14.24) |
| *Personalized services according to the genetic profile of each individual can be provided* | 8 (2.65) | 39 (12.91) | 72 (23.84) | 135 (44.70) | 48 (15.89) |
| *Consumers can undergo tests in private* | 26 (8.61) | 70 (23.18) | 98 (32.45) | 82 (27.15) | 26 (8.61) |
| *Raise awareness about genetics* | 11 (3.64) | 25 (8.28) | 81 (26.82) | 133 (44.04) | 52 (17.22) |
| *Individuals can learn about genetic conditions they may be at risk for* | 9 (2.98) | 28 (9.27) | 64 (21.19) | 145 (48.01) | 56 (18.54) |
| *Encouraging individuals to take responsibility for their health* | 11 (3.64) | 38 (12.58) | 78 (25.83) | 114 (37.75) | 61 (20.20) |
| *Stimulating individuals to facilitate family discussions surrounding their personal health* | 9 (2.98) | 33 (10.93) | 99 (32.78) | 115 (38.08) | 46 (15.23) |
| *Positive impact on the patient-doctor relationship* | 31 (10.26) | 67 (22.19) | 108 (35.76) | 71 (23.51) | 25 (8.28) |

**Table S6**. Predictors of knowledge and awareness of healthcare professionals about DTC-GT (ORadj: adjusted Odds Ratio; OR: Odds Ratio; CI: 95% confidence interval)

|  |  | **Knowledge** | | **OR (CI)** | **p-value** | **ORadj (CI)** | **p-value** |
| --- | --- | --- | --- | --- | --- | --- | --- |
|  |  | **No** | **Yes** |  |  |  |  |
| *Sex* | Female | 117 (70.1) | 50 (29.9) | - |  |  |  |
|  | Male | 101 (74.8) | 34 (25.2) | 0.78 (0.47-1.31) | 0.36 |  |  |
| *Age* |  | 35 (30-44) | 31 (28-42) | 0.98 (0.96-1.01) | 0.361 |  |  |
| *History of genetic/ hereditary disorders* | No | 196 (73.4) | 71 (26.6) | - |  |  |  |
|  | Yes | 22 (62.9) | 13 (37.1) | 1.63 (0.78-3.41) | 0.193 |  |  |
| *History of cancer* | No | 117 (72.7) | 44 (27.3) | - |  |  |  |
|  | Yes | 101 (71.6) | 40 (28.4) | 1.05 (0.63-1.74) | 0.841 |  |  |
| *Genetics/ genomics involvement* | No | 162 (80.2) | 40 (19.8) | - |  |  |  |
|  | Yes | 56 (56) | 44 (44) | 3.18 (1.88-5.37) | <0.0001 | 2.90 (1.70-4.95) | <0.0001 |
| *Degree area* | Non-Medicine | 98 (76.6) | 30 (23.4) | - |  |  |  |
|  | Medicine | 120 (69) | 54 (31) | 1.47 (0.87-2.47) | 0.146 | 1.14 (0.64-2.05) | 0.637 |
| *Post- or undergraduate training* | No | 71 (84.5) | 13 (15.5) | - |  |  |  |
|  | Yes | 85 (72) | 33 (28) | 2.63 (1.36-5.08) | 0.004 | 2.09 (1.02-4.30) | 0.043 |
| *Work sector* | Non-Academic | 115 (74.2) | 40 (25.8) | - |  |  |  |
|  | Academic | 103 (70.1) | 44 (29.9) | 1.22 (0.74-2.03) | 0.424 |  |  |

**Table S7**. Predictors of attitudes of healthcare professionals towards DTC-GT (ORadj: adjusted Odds Ratio; OR: Odds Ratio; CI: 95% confidence interval)

|  |  | **Attitude** | | **OR (CI)** | **p-value** | **ORadj (CI)** | **p-value** |
| --- | --- | --- | --- | --- | --- | --- | --- |
|  |  | **No** | **Yes** |  |  |  |  |
| *Sex* | Female | 111 (66.5) | 56 (33.5) | - |  |  |  |
|  | Male | 88 (65.2) | 47 (34.8) | 1.05 (0.65-1.70) | 0.815 |  |  |
| *Age* |  | 35 (30-45) | 31 (28-41) | 0.96 (0.94-0.99) | 0.011 | 0.97 (0.95-1.00) | 0.063 |
| *History of genetic/ hereditary disorders* | No | 179 (67) | 88 (33) | - |  |  |  |
|  | Yes | 20 (57.1) | 15 (42.9) | 1.52 (0.74-3.12) | 0.248 |  |  |
| *History of cancer* | No | 105 (65.2) | 56 (34.8) | - |  |  |  |
|  | Yes | 94 (66.7) | 47 (33.3) | 0.93 (0.58-1.51) | 0.791 |  |  |
| *Genetics/ genomics involvement* | No | 133 (65.8) | 69 (34.2) | - |  |  |  |
|  | Yes | 66 (66) | 34 (34) | 0.99 (0.59-1.64) | 0.978 |  |  |
| *Degree area* | Non-Medicine | 98 (76.6) | 30 (23.4) | - |  |  |  |
|  | Medicine | 101 (58) | 73 (42) | 2.36 (1.42-3.92) | 0.001 | 2.07 (1.23-3.50) | 0.006 |
| *Post- or undergraduate training* | No | 60 (71.4) | 24 (28.6) | - |  |  |  |
|  | Yes | 84 (71.2) | 34 (28.8) | 1.42 (0.82-2.45) | 0.209 |  |  |
| *Work sector* | Non-Academic | 101 (65.2) | 54 (34.8) | - |  |  |  |
|  | Academic | 98 (66.7) | 49 (33.3) | 0.93 (0.58-1.50) | 0.783 |  |  |
| *Knowledge* | No | 153 (70.2) | 65 (29.8) | - |  |  |  |
|  | Yes | 46 (54.8) | 38 (45.2) | 1.94 (1.15-3.26) | 0.012 | 1.82 (1.07-3.10) | 0.027 |

**Table S8**. Predictors of favourable behaviour of healthcare professionals towards DTC-GT (ORadj: adjusted Odds Ratio; OR: Odds Ratio; CI: 95% confidence interval)

|  |  | **Behaviour** | | **OR (CI)** |  | **ORadj (CI)** |  |
| --- | --- | --- | --- | --- | --- | --- | --- |
|  |  | **No** | **Yes** |  |  |  |  |
| *Sex* | Female | 154 (92.2) | 13 (7.8) | - |  |  |  |
|  | Male | 129 (95.6) | 6 (4.4) | 0.55 (0.20-1.49) | 0.240 |  |  |
| *Age* |  | 35 (30-45) | 31 (28-41) | 0.98 (0.94-1.03) | 0.550 |  |  |
| *History of genetic/ hereditary disorders* | No | 252 (94.4) | 15 (5.6) | - |  |  |  |
|  | Yes | 31 (88.6) | 4 (11.4) | 2.16 (0.67-6.94) | 0.193 |  |  |
| *History of cancer* | No | 149 (92.5) | 12 (7.5) | - |  |  |  |
|  | Yes | 134 (95) | 7 (5) | 0.64 (0.24-1.69) | 0.377 |  |  |
| *Genetics/ genomics involvement* | No | 189 (93.6) | 13 (6.4) | - |  |  |  |
|  | Yes | 94 (94) | 6 (6) | 0.92 (0.34-2.51) | 0.883 |  |  |
| *Degree area* | Non-Medicine | 119 (93) | 9 (7) | - |  |  |  |
|  | Medicine | 164 (94.3) | 10 (5.7) | 0.80 (0.31-2.04) | 0.650 |  |  |
| *Post- or undergraduate training* | No | 80 (95.2) | 4 (4.8) | - |  |  |  |
|  | Yes | 203 (93.1) | 15 (6.9) | 1.47 (0.47-4.58) | 0.499 |  |  |
| *Work sector* | Non-Academic | 143 (92.3) | 12 (7.7) | - |  |  |  |
|  | Academic | 140 (95.2) | 7 (4.8) | 0.59 (0.22-1.55) | 0.291 |  |  |
| *Knowledge* | No | 203 (93.1) | 15 (6.9) | - |  |  |  |
|  | Yes | 80 (95.2) | 4 (4.8) | 0.67 (0.21-2.10) | 0.499 |  |  |
| *Attitude* | No | 186 (93.5) | 13 (6.5) | - |  |  |  |
|  | Yes | 97 (94.2) | 6 (5.8) | 0.88 (0.32-2.40) | 0.810 |  |  |
